# Regulation of Mcl-1 by constitutive activation of NF-kappaB contributes to cell viability in human esophageal squamous cell carcinoma cells

**DOI:** 10.1186/1471-2407-14-98

**Published:** 2014-02-17

**Authors:** Haidan Liu, Jinfu Yang, Yunchang Yuan, Zhenkun Xia, Mingjiu Chen, Li Xie, Xiaolong Ma, Jian Wang, Sufeng Ouyang, Qin Wu, Fenglei Yu, Xinmin Zhou, Yifeng Yang, Ya Cao, Jianguo Hu, Bangliang Yin

**Affiliations:** 1 Department of Cardiothoracic Surgery, The Second Xiangya Hospital, Central South University, 139 Renmin Road, Changsha, Hunan 410011, China; 2 Clinical Center for Gene Diagnosis and Therapy, The Second Xiangya Hospital, Central South University, 139 Renmin Road, Changsha, Hunan 410011, China; 3 Cancer Research Institute, Xiangya School of Medicine, Central South University, 110 Xiangya Road, Changsha, Hunan 410078, China

**Keywords:** Esophageal squamous cell carcinoma, Gene regulation, NF-κB, Mcl-1, Cell viability

## Abstract

**Background:**

Esophageal squamous cell carcinoma (ESCC) is one of the most lethal malignancies with a 5-year survival rate less than 15%. Understanding of the molecular mechanisms involved in the pathogenesis of ESCC becomes critical to develop more effective treatments.

**Methods:**

Mcl-1 expression was measured by reverse transcription (RT)-PCR and Western blotting. Human *Mcl-1* promoter activity was evaluated by reporter gene assay. The interactions between DNA and transcription factors were confirmed by electrophoretic mobility shift assay (EMSA) *in vitro* and by chromatin immunoprecipitation (ChIP) assay in cells.

**Results:**

Four human ESCC cell lines, TE-1, Eca109, KYSE150 and KYSE510, are revealed increased levels of Mcl-1 mRNA and protein compare with HaCaT, an immortal non-tumorigenic cell line. Results of reporter gene assays demonstrate that human *Mcl-1* promoter activity is decreased by mutation of kappaB binding site, specific NF-kappaB inhibitor Bay11-7082 or dominant inhibitory molecule DNMIkappaBalpha in TE-1 and KYSE150 cell lines. Mcl-1 protein level is also attenuated by Bay11-7082 treatment or co-transfection of DNMIkappaBalpha in TE-1 and KYSE150 cells. EMSA results indicate that NF-kappaB subunits p50 and p65 bind to human Mcl-1-kappaB probe *in vitro.* ChIP assay further confirm p50 and p65 directly bind to human *Mcl-1* promoter in intact cells, by which regulates Mcl-1 expression and contributes to the viability of TE-1 cells.

**Conclusions:**

Our data provided evidence that one of the mechanisms of Mcl-1 expression in human ESCC is regulated by the activation of NF-kappaB signaling. The newly identified mechanism might provide a scientific basis for developing effective approaches to treatment human ESCC.

## Background

Human esophageal squamous cell carcinoma (ESCC) is one of the most frequently diagnosed carcinomas, ranked as the sixth leading cause of death from cancers worldwide. ESCC remains the most common histology and occurs at a very high frequency in China, South Africa, France and Italy [1]. Although modest advances have been made in chemotherapy for esophageal cancer, ESCC is still one of the most aggressive types of cancer with a 5-year survival rate less than 15%. The underlying reasons for this disappointingly low survival rate remains to be greatly elucidated. Therefore, a better understanding of the molecular mechanisms of ESCC pathogenesis is expected to facilitate the development of novel therapies for this disease.

The *Mcl-1* is an antiapoptotic gene of the Bcl-2 family members. Mcl-1 is overexpressed in many human tumor specimens, including hepatocellular carcinoma [2], pancreatic cancer [3], prostate cancer [4] and others [5]. Overexpression of Mcl-1 was found in malignant melanoma compared to benign nevi and increased expression of Mcl-1 was also observed by comparing primary and metastatic melanoma samples utilizing a tissue microarray [6]. In addition, frequent *Mcl-1* gene amplification was identified in lung, breast, neural and gastrointestinal cancers, through which cancer cells depend on the expression of this gene for survival [7]. A survey of antiapoptotic Bcl-2 family member expression in breast, brain, colon, lung, ovarian, renal and melanoma cell lines revealed that *Mcl-1* mRNA is more abundant than Bcl-2 or Bcl-xL [8]. These studies demonstrated that Mcl-1 plays a critical role in carcinogenesis and malignancy development in a broad range of human tumors, making it an attractive therapeutic target. However, the underlying mechanisms causing its elevation are not fully understood.

Expression of *Mcl-1* gene can be regulated at transcriptional level. Analysis of human *Mcl-1* gene 5′-flanking promoter regions for potential transcription factor binding sites revealed consensus sequences including STAT, SRE, Ets, Sp1, CRE-BP [9]. Multiple intracellular signaling pathways and transcription factors have been confirmed to influence Mcl-1 expression, including PI3K/Akt [10], Stat3 [11,12], CREB [10], Ets family members Elk-1 [13] and PU.1 [14]. In addition, putative binding sites for NF-κB were identified in the *Mcl-1* promoter region [9]. Previous studies demonstrated that inhibition of NF-κB activation by a novel NF-κB inhibitor V1810 [15] or Thiocolchicoside [16] accompanied by the downregulation of Mcl-1 expression. However, the underlying mechanistic link between NF-κB and Mcl-1 expression has not been clearly established in these studies. Moreover, although reports [17,18] have revealed that p65 subunit of NF-κB involves in TRAIL induced expression of Mcl-1 in HCT-116 colon carcinoma cells [17] and the interaction of p65 with N-a-Acetyltransferase 10 protein regulates Mcl-1 expression [18], the precise mechanism of *Mcl-1* transcriptionally controlled by NF-κB family members is not fully elucidated. Therefore, a better understanding the role of this regulatory molecule in Mcl-1 expression in cancers may allow for the development of rational therapeutics that control Mcl-1 levels.

Transcripition factor NF-κB comprised of homo- and heterodimers of the RelA (p65), RelB, c-Rel, p50/p105 (NF-κB1) and p52/p100 (NF-κB2) polypeptides can both induce and repress gene expression by binding to discrete κB elements in promoters and enhancers. The genes regulated by NF-κB include those controlling apoptosis, cell adhesion, proliferation, and inflammation. In most untransformed cell types, NF-κB complexes are largely cytoplasmic by a family of inhibitory proteins known as inhibitors of NF-κB (IκBs) and therefore remain transcriptionally inactive [19]. Activation of NF-κB typically involves the phosphorylation of IκB by the IκB kinase (IKK) complex, which results in IκB degradation. This liberates NF-κB and allows it to translocate freely to the nucleus and binds to the κB elements in the relevant downstream genes to activate a series of transcriptional events [19]. It has become apparent that aberrant activation of NF-κB in human cancers are common [20]. Activation of NF-κB has been detected in tumor samples from patients, such as breast, colorectal, ovarian, pancreatic, prostate cancers and so forth [21,22]. Constitutive NF-κB activation has also reported in esophageal carcinoma tissues [22,23] and cell lines [24], implying NF-κB activation plays an important role in the tumorigenesis and development of human ESCC. Expression of Mcl-1 has been shown in human esophageal carcinoma cell lines CE81T/VGH [25] and KYSE450 [26]. We thus speculated that a direct link might exist between NF-κB and Mcl-1 expression in human ESCC.

The present study was performed to determine whether Mcl-1 expression is modulated by NF-κB signal pathway in human ESCC. Using human ESCC cell lines as models, reporter gene assays demonstrate that human *Mcl-1* promoter activity is decreased by mutation of κB site, specific NF-κB inhibitor Bay11-7082 or dominant inhibitory molecule DNMIκBα in TE-1 and KYSE150 cells. Mcl-1 level is attenuated by Bay11-7082 treatment or co-transfection of DNMIκBα in TE-1 and KYSE150 cells. NF-κB subunits p50 and p65 are further confirmed bound to Mcl-1-κB probe *in vitro* by EMSA assay and directly bound to human *Mcl-1* promoter in intact cells by ChIP assay, respectively. Our data provided evidence that one of the regulatory mechanisms by which Mcl-1 expression in human ESCC is by binding of p50 and p65 to κB site within human *Mcl-1* promoter. This NF-κB mediating Mcl-1 expression also contributes to the viability of TE-1 cells. In conclusion, the newly identified mechanism might provide a scientific basis for developing effective approaches to treatment human ESCC.

## Methods

### Cell lines and culture

Human esophageal carcinoma cell lines TE-1 and Eca109 were purchased from Cell Bank of Chinese Academy of Sciences, Shanghai, China. Human esophageal carcinoma cell lines KYSE150 and KYSE510 were kindly provided by Dr. Qian Tao from The Chinese University of Hong Kong, HongKong, China. Immortalized human keratinocyte cell line HaCaT derived from human adult trunk skin was previous described [27,28]. TE-1, Eca109, KYSE150 and KYSE510 cells were cultured in RPMI 1640 medium (Invitrogen, Carlsbad, CA) supplemented with 10% fetal bovine serum, 100 units/ml penicillin and 100 mg/ml streptomycin. HaCaT was cultured in DMEM medium (Invitrogen, Carlsbad, CA) containing 10% fetal bovine serum and antibiotics as described above. All cell lines were incubated at 37˚C in a humidified atmosphere containing 5% CO2.

### Chemicals and cell treatments

The specific NF-κB inhibitor Bay11-7082 (Calbiochem, Darmstadt, Germany) was prepared as a stock solution of 20 mM in DMSO (Sigma, St. Louis, MO). Subconfluent cells were treated with the compound at indicated concentrations for an indicated time. Detailed treatment procedures were described in figure legends. The final concentration of DMSO in the culture media was kept less than 0.1% which had no significant effect on the cell growth. Vehicle controls were prepared for all treatments.

### Plasmids

The pGL2-Mcl-1-κBwt (Addgene plasmid 19132) which contains a 325 bp long human *Mcl-1* promoter fragment including NF-κB binding-site (GGGGTCTTCC) and the pGL2-Mcl-1-κBmt (Addgene plasmid 19133) in which the κB site sequence GGGGTCTTCC being changed to GTTGTCTTCC were constructed by Dr. El-Deiry [17] and obtained through Addgene (Cambridge, MA). The pGL2-Basic vector was purchased from Promega (Madison, WI). The pGL3-Basic vector and pGL3-NF-κB-Luc were the same as described previously [29,30]. Expression plasmid of dominant negative mutant of IκBα (pcDNA3-DNMIκBα) [30] and the pcDNA3.1 empty vector [31] were identical to those used previously. The human full-length Mcl-1 expression vector pCMV6-A-Puro-Mcl and pCMV6-A-Puro empty vector were kindly provided by Dr. Chengchao Shou [18].

### Transfection and luciferase reporter assays

Cells were cultured in 24-well plates at a density of 1 × 10^5^ per well overnight and transfected with Lipofectamine™ 2000 (Invitrogen, Carlsbad, CA) according to manufacturer’s instructions. In luciferase assay for NF-κB transactivation, each transfection contained 800 ng/well of pGL3-Basic or pGL3-NF-κB-Luc together with 40 ng/well of internal control pRL-SV40 (Promega, Madison, WI) (Total DNA 840 ng/well). 24 h after transfection, cells were either left untreated (DMSO) or treated with 20 μM Bay11-7082 for 12 h. Cells were harvested at 36 h after transfection and lysates were analyzed for luciferase activity using the Dual Luciferase Reporter assay (Promega, Madison, WI) with a GloMax™ Microplate Luminometer (Promega, Madison, WI). In luciferase assay for the *Mcl-1* promoter, each transfection contained 400 ng/well of pGL2-Basic, pGL2-Mcl-1-κBwt or pGL2-Mcl-1-κBmt together with 400 ng/well of pcDNA3.1 or pcDNA3-DNMIκBα expression plasmid. Each transfection contained 40 ng/well of pRL-SV40 as internal control (Total DNA 840 ng/well). 24 h after transfection, cells were either left untreated (DMSO) or treated with 20 μM Bay11-7082 for 12 h. Cells were harvested at 36 h after transfection and lysates were analyzed as described above. The pRL-SV40 was co-transfected in all experiments to correct the variations in transfection efficiency. The data represent the mean ± S.D. of at least two independent experiments performed in triplicate.

### RNA interference

TE-1 cells were grown in 6-well plates at a density of 3 × 10^5^ cells per well overnight. Cells reached 60-70% confluency on the day of transfection and were transfected with a p50 (sc-29407; 100 pmol), a p65 (sc-29410; 100 pmol) or a scrambled control (sc-37007; 100 pmol) siRNA (all from Santa Cruz Biotechnology) using HiPerFect transfection reagent (Cat no: 301705, Qiagen) for 72 h according to the manufacturer’s instructions. Cells were harvested for protein extraction and immunoblotting to confirm p50 or p65 knockdown.

### Cell viability assay

Cell viability assays were performed using the 4-[3-(4-iodophenyl)-2-(4-nitrophenyl)-2H-5-tetrazolio]-1,3-benzene disulfonate (WST-1) assay kit (Roche, Indianapolis, IN) according to the manufacturer’s instructions. The assay is based on the cleavage of WST-1 to formazan dye by cellular mitochondrial dehydrogenases. Because cleavage of WST-1 to formazan dye occurs only in viable cells, the amount of dye produced, measured in OD values, directly corresponds with the number of viable cells present in the culture. Briefly, TE-1 cells were firstly transfected with the control, p50 or p65 siRNA in six-well plates as described above. To investigate whether reintroduction of Mcl-1 restored cell viability, 24 h following the first transfection, a second transient transfection was carried out to ectopically express Mcl-1. Each transfection contained 2 μg pCMV6-A-Puro empty vector or pCMV6-A-Puro-Mcl construct using SuperFect transfection reagent (Cat no: 301305, Qiagen) according to the manufacturer’s instructions. At 24 h post-transfection, cells were trypsinized, an aliquot of cells was maintained in six-well plate, harvested at 120 h after NF-κB subunit siRNA transfection and analyzed the Mcl-1 levels by Western blotting. The remainder was transferred as six replicates to 96-well plates at a concentration of 2.5 × 10^3^ cells per well in 100 μl of complete RPMI 1640. After culturing for another 24, 48, 72 h (i.e. 72 h, 96 h, 120 h after each siRNA transfection, respectively), 10 μl of WST-1 was added to each well and cells incubated for 2 h at 37°C. The cellular reduction of WST-1 to formazan and its absorbance were measured at 450 nm.

### Protein preparation and western blotting

Cultured cells were harvested and whole cell lysates were prepared according to the method previously described [30]. Nuclear extracts were prepared using a Nuclear Extract kit (Cat. no. 40010, Active Motif, Carlsbad, CA) following the manufacturer’s instructions. Protein concentration was determined using the BCA Assay Reagent (Cat. no. 23228, Pierce, Rockford, IL). Western blotting was performed as previously described [30]. The following antibodies were used for immunodetection with appropriate dilutions: Mcl-1 (sc-819, 1:1000), p50 (sc-114, 1:1000), p52 (sc-298, 1:1000), p65 (sc-8008, 1:1000), c-Rel (sc-272, 1:1000), RelB (sc-226, 1:1000) and GAPDH (sc-47724, 1:2000) (all from Santa Cruz, CA); Histone H3 (#9715, 1:1000) were purchased from Cell Signaling Technology (Beverly, MA); β-actin (A5316, 1:5000) was purchased from Sigma (St. Louis, MO).

### mRNA extraction and reverse transcription-polymerase chain reaction (RT-PCR)

Total RNA was extracted using Trizol reagent (Invitrogen, Carlsbad, CA). First-strand cDNA was synthesized from 2 μg of total RNA using the Reverse Transcription System Kit (Cat. No. A3500, Promega, Madison, WI). The resulted cDNA was subjected to PCR (94°C for 5 min followed by 34 cycles of 94°C for 30 s, 58°C for 30 s, 72°C for 40 s, and an extension for 10 min at 72°C) using primers designed for human Mcl-1 [11]: sense, 5′-cggcagtcgctggagattat-3′ and antisense, 5′-gtggtggtggttggtta-3′, yield a 573-bp product; or for GAPDH: sense, 5′-caaagttgtcatggatgacc-3′ and antisense, 5′-ccatggagaaggctgggg-3′, yield a 195-bp product. Real-time RT-PCR experiments were done in triplicate as described previously [32] and the primers used were as following [33]: forward 5′-gggcaggattgtgactctcatt-3′; reverse 5′-gatgcagctttcttggtttatgg-3′. The relative Mcl-1 mRNA expression levels were calculated according to the comparative CT (∆∆CT) method after normalizing to GAPDH expression. Semiquantitive RT-PCR products were separated on 1.5% agarose gels and visualized with ethidium bromide. The identity of Mcl-1 PCR product was confirmed by direct sequencing after purification.

### Electrophoretic mobility shift assays

Nuclear proteins from cultured cells were prepared and protein concentration was determined as described above. EMSA was performed using the LightShift™ Chemiluminescent EMSA Kit (Cat. No. 20148, Pierce, Rockford, IL) following the manufacturer’s instructions. The reaction mixtures (20 μl) containing 8 μg nuclear extracts were incubated with 2 nM of biotin-labeled double-stranded oligonucleotide probes in reaction buffer for 20 min at room temperature. Samples were subjected to electrophoresis in 5% nondenaturing polyacrylamide gel and transferred to Biodyne™ BNylon membrane (Cat. No. 77016, Pierce, Rockford, IL). For competition analyses, 100-fold excess of unlabeled probes were included in the binding reaction. For antibody supershift experiments, the reaction mixtures were preincubated with 2 μg of p50 (sc-8414X), p52 (sc-298X), p65 (sc-8008X), c-Rel (sc-272X), RelB (sc-226X) or rabbit IgG (sc-2027) antibody (all from Santa Cruz, CA) for 30 min at room temperature. Biotin-labeled double-stranded oligonucleotides were used as probes listed below: wild-type NF-κB consensus binding sequence: 5′-agttgag*gggactttcc*caggc-3′ [34]; wild-type Mcl-1-κB binding sequence: 5′-ggagtc*ggggtcttccc*cagtttt-3′, corresponding to the nucleotides of the human *Mcl-1* promoter. Unlabeled double-stranded oligonucleotides used for competition analyses were: wild-type NF-κB consensus binding sequence: 5′-agttgag*gggactttcc*caggc-3′; mutated NF-κB consensus binding sequence: 5′-agttgag*gagatctggc*caggc-3′ [34]; mutant Mcl-1-κB binding sequence: 5′-ggagtc*g* ***tt*** *gtcttcc*ccagtttt-3′; The AP-1 consensus probe was used as a nonspecific competitor for NF-κB: 5′-cgcttga*tgagtca*gccggaa-3′ [35]. The probes were commercially synthesized by TaKaRa Bio Inc. (Dalian, China). Binding sites were indicated in italics type and mutations were shown in bold type. The mutated nucleotides for NF-κB binding site of human *Mcl-1* promoter in EMSA were identical to those of the mutated sequences in the reporter construct.

### Chromatin immunoprecipitation (ChIP) assay

ChIP was performed using the ChIP assay kit (Upstate Biotechnology, Lake Placid, NY) as previously described [30]. Antibodies used for immunoprecipitation were: p50 (sc-8414X), p52 (sc-298X), p65 (sc-8008X), c-Rel (sc-272X), RelB (sc-226X) and rabbit IgG (sc-2027) (all from Santa Cruz, CA). 2 μg of each antibody was used for each immunoprecipitation. The following primers were used in the ChIP assays: human *Mcl-1* promoter including the NF-κB binding region, 5′-cacttctcacttccgcttcc-3′ and 5′-ttctccgtagccaaaagtcg-3′ (200 bp).

### Statistical analysis

Statistical analysis was done with the statistical software program SPSS ver.12.0. Results expressed as mean ± S.D. were analyzed using the Student’s *t* test. Differences were considered significant when P value was <0.05.

## Results

### Expression of Mcl-1 mRNA and protein in human esophageal squamous cell carcinoma cell lines

**Figure 1:**
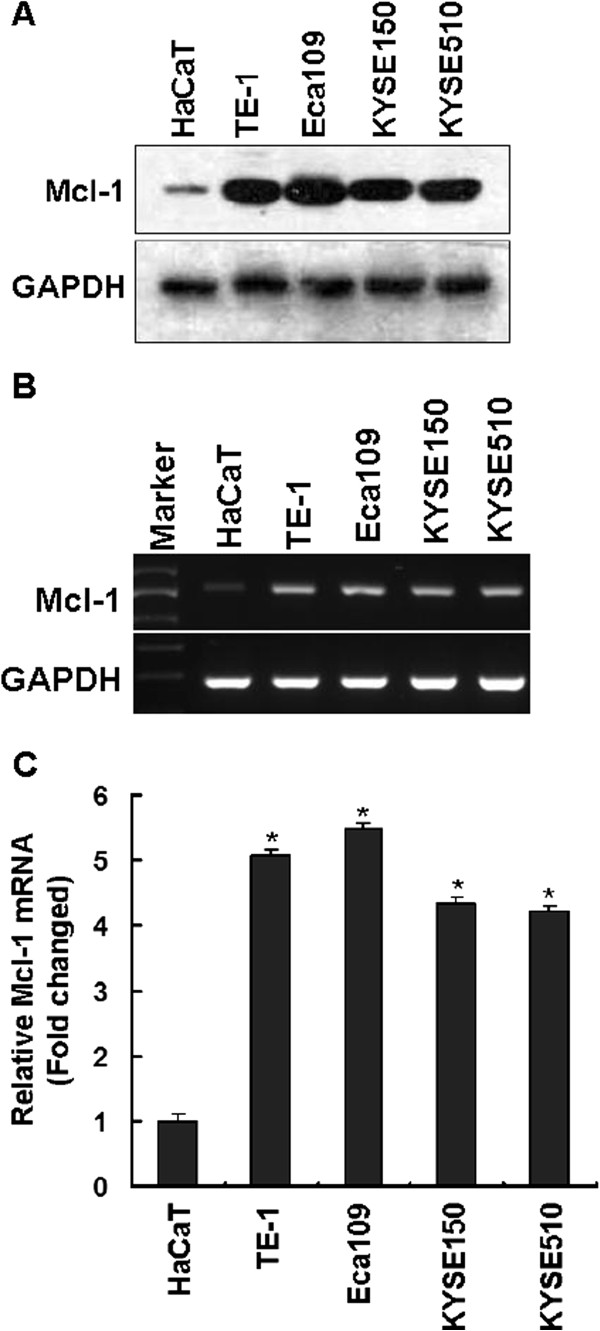
**Expression of Mcl-1 protein and mRNA in human esophageal squamous cell carcinoma cell lines. (A)** Expression of Mcl-1 protein in various esophageal carcinoma cell lines. Whole cell lysates of TE-1, Eca109, KYSE150 and KYSE510 as well as HaCaT cell lines were subjected to immunoblotting analysis with anti-Mcl-1 antibody. GAPDH was used as a loading control. **(B)** Total RNA was isolated from above-mentioned cell lines and subjected to RT-PCR, using specific primers designed to amplify Mcl-1 and GAPDH mRNAs. GAPDH was used as a loading control. **(C)***Mcl-1* mRNA expression levels of TE-1, Eca109, KYSE150 and KYSE510 as well as HaCaT cell lines were analyzed by quantitative real-time RT-PCR. The *Mcl-1* mRNA level of HaCaT cells was normalized to a value of 1. Fold-change in mRNA levels was shown. Data are presented as the mean ± S.D. of two independent experiments performed in triplicate. Statistical significance: *, p < 0.01, compared with HaCaT cells.

To investigate the expression patterns of Mcl-1 in human ESCC cell lines, Mcl-1 expression was first measured by Western blotting. As shown in Figure 1A, four human esophageal carcinoma cell lines, including TE-1, Eca109, KYSE150 and KYSE510 revealed increased levels of Mcl-1 protein compare with an immortal non-tumorigenic keratinocyte HaCaT cell line [27], which was used as a normal control [36,37] for Mcl-1 expression. The Mcl-1 protein levels among these esophageal carcinoma cell lines were similar (Figure 1A). In addition, semi-quantitative RT-PCR was performed to analyze the *Mcl-1* mRNA expression in these cell lines. The RT-PCR results indicated increased expression of *Mcl-1* mRNA levels in four human ESCC cell lines compared with that in HaCaT cells (Figure 1B), which was in agreement with the observations in the immunoblotting analysis. We also performed quantitative real-time RT-PCR to compare mRNA levels of *Mcl-1* in these cell lines. As shown in Figure 1C, higher mRNA levels of *Mcl-1* in TE-1, Eca109, KYSE150 and KYSE510 cells, about a 5-fold increase of *Mcl-1* for each cell line compared with HaCaT cells. The observations that Mcl-1 protein levels corresponding exactly with its mRNA levels suggested Mcl-1 expression was regulated, at least in part, at transcriptional level in human ESCC cells.

### NF-κB is constitutively activated in Mcl-1-expressing human esophageal squamous cell carcinoma cell lines

**Figure 2:**
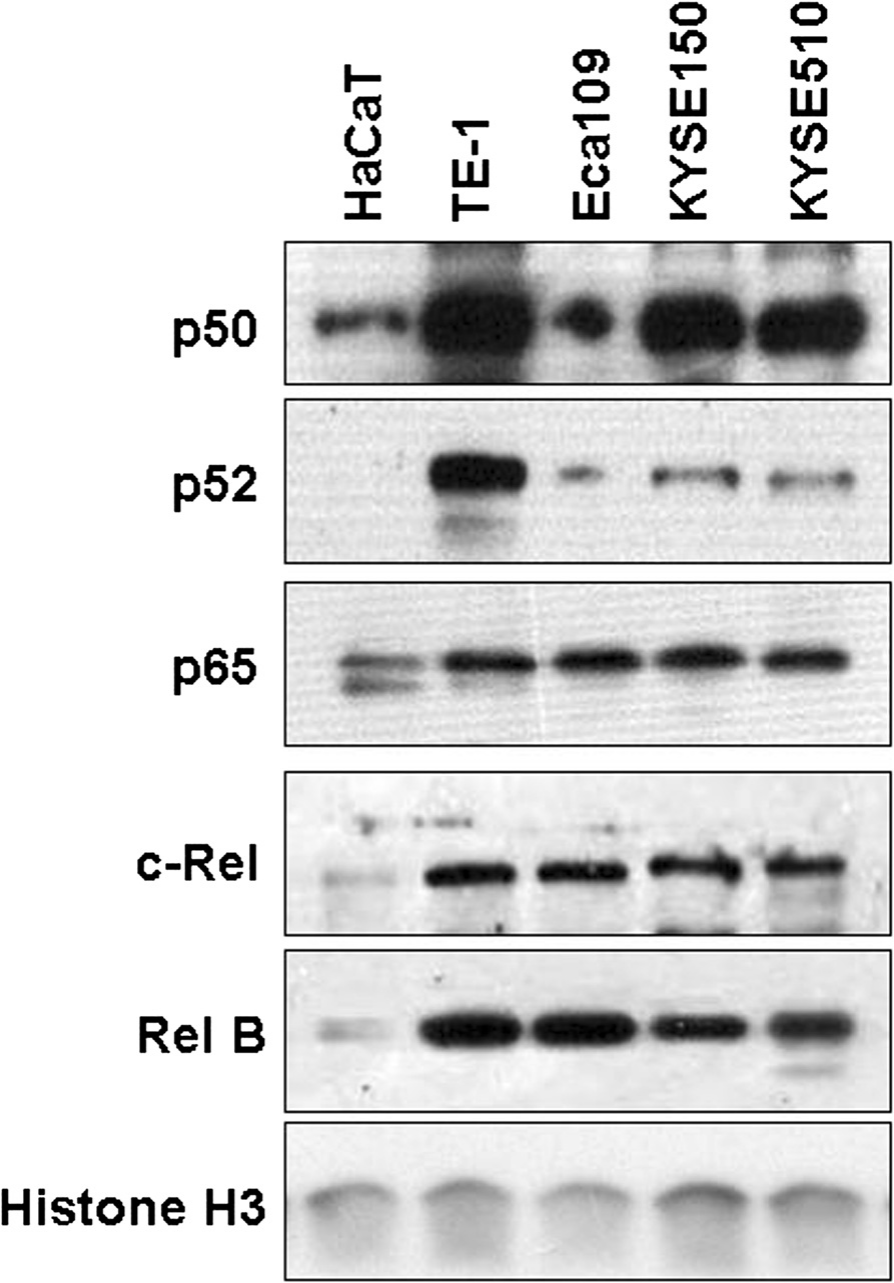
**Nucleus distribution of NF-κB family members in human esophageal squamous cell carcinoma cell lines.** Nuclear extracts of TE-1, Eca109, KYSE150, KYSE510 and HaCaT were prepared as described in Methods and analyzed with antibodies against NF-κB subunits p50, p52, p65, c-Rel and RelB, respectively. Histone H3 was used as a nuclear protein loading control.

NF-κB has been shown to play a role in TRAIL-induced Mcl-1 expression in HCT-116 colon cancer cells [17] and the interaction of p65 subunit with Naa10p reportedly regulates Mcl-1 expression [18], However, whether NF-κB is involved in Mcl-1 expression in human ESCC cells remains to be clarified. To address this issue, we initially evaluated whether NF-κB is constitutively activated in Mcl-1-expressing human ESCC cells. NF-κB activation as measured by nuclear accumulation has been observed in a wide variety of solid tumors [22]. Therefore, nuclear extracts of TE-1, Eca109, KYSE150, KYSE510 and HaCaT cell lines and the levels of NF-κB subunits in nucleus were estimated. Histone H3 level served as a loading control for nuclear protein [38]. The levels of NF-κB subunits in nuclear extracts of four ESCC cell lines were markedly higher than those in HaCaT cells, suggested that NF-κB is highly constitutively activated in these ESCC cell lines detected. The results indicated that TE-1 cell line displayed relatively high levels of NF-κB subunit p50 and p52. The expression patterns of NF-κB subunit p65, c-Rel and RelB were similar in other three esophageal carcinoma cell lines (Figure 2). The distinctive patterns for constitutively activated NF-κB subtypes in different ESCC cell lines suggested that NF-κB subunits might play a specific role in regulating Mcl-1 in different esophageal carcinoma cell lines. These results led to the conclusion that the NF-κB pathway is constitutively activated in Mcl-1-expressing human ESCC cell lines.

### The role for NF-κB signaling pathway in regulating the *Mcl-1* promoter activity in various human esophageal squamous cell carcinoma cell lines

**Figure 3:**
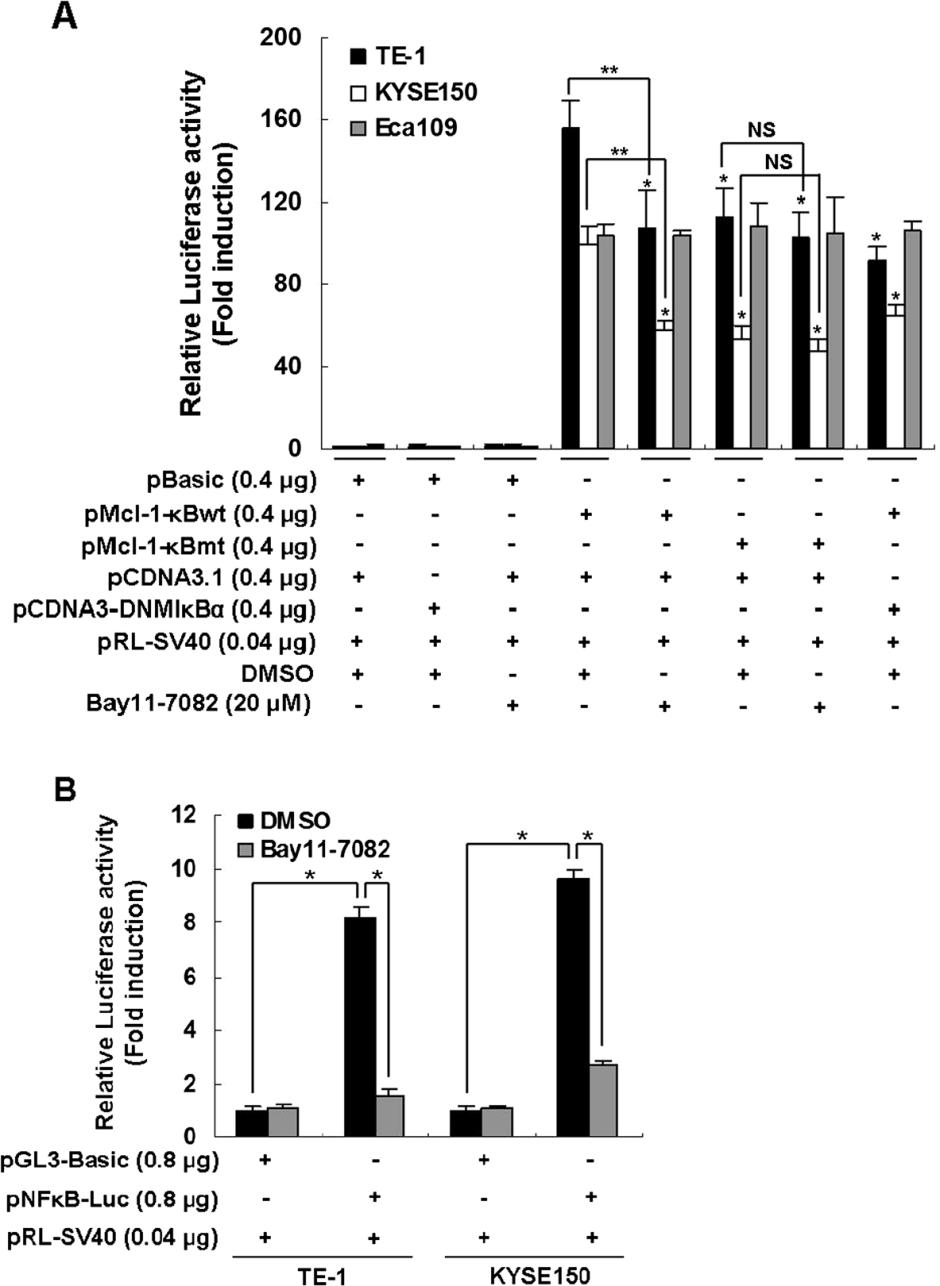
**The role for NF-κB signaling pathway in regulating transcriptional activity of human *Mc1-1 *promoter in various human esophageal squamous cell carcinoma cell lines. (A)** TE-1, KYSE150 or Eca109 cells were transfected of pGL2-Basic, pMcl-1-κBwt or pMcl-1-κBmt together with pcDNA3.1 empty vector or pcDNA3-DNMIκBα plasmid. The pRL-SV40 was co-transfected in all experiments to correct the variations in transfection efficiency. Transfected cells were incubated for 24 h and then treated with DMSO or 20 μM Bay11-7082 for an additional 12 h after which the activities of firefly and Renilla luciferase were monitored by dual luciferase reporter assay. The relative luciferase activity normalized to the value of Renilla luciferase activity. The *Mcl-1* promoter activities were expressed as fold induction of their respective pGL2-Basic-transfected-cells treated with DMSO. Data are shown as means ± S.D. of at least two independent experiments performed in triplicate. Statistical significance: * p < 0.05, compared with their respective pMcl-1-κBwt-transfected-cells treated with DMSO. ** p < 0.05, pMcl-1-κBwt-transfected-cells treated with DMSO versus pMcl-1-κBwt-transfected-cells treated with Bay11-7082. NS, no significant difference between pMcl-1-κBmt-transfected-cells treated with DMSO and pMcl-1-κBmt-transfected-cells treated with Bay11-7082. **(B)** Effects of Bay11-7082 on the transactivation activity of NF-κB in TE-1 and KYSE150 cell lines. TE-1 or KYSE150 cells were transient transfected with pGL3-Basic vector or pGL3-NF-κB-Luc plasmid and luciferase reporter assay were performed as described in Methods. The relative luciferase activity normalized to the value of Renilla luciferase activity. NF-κB-driven luciferase activities were expressed as fold induction of their respective pGL3-Basic-transfected-cells treated with DMSO. Data are presented as the mean ± S.D. of two independent experiments performed in triplicate. Statistical significance: *, p < 0.01.

To examine whether NF-κB activated transcription from the promoter of human *Mcl-1* gene in Mcl-1-expressing ESCC cell lines, different series of human esophageal carcinoma cell lines TE-1, Eca109 and KYSE150 were transiently transfected with the luciferase reporter plasmid containing a 325 bp long human *Mcl-1* promoter fragment. As seen in Figure 3A, transfection of the pGL2-Mcl-1-κBwt generated higher luciferase activity than that of the pGL2-Basic construct, indicated that high transcriptional activity of human *Mcl-1* promoter in three Mcl-1-expressing ESCC cell lines tested. However, with a promoter construct mutated at the κB site, the loss of *Mcl-1* promoter activity was observed in TE-1 and KYSE150 cells (Figure 3A). Dominant negative mutants of IκBα (DNMIκBα), a truncant mutant with a deletion of 71 amino acids at the N terminus of IκBα, can competitively inhibit the activation of NF-κB was used to block NF-κB activation as described previously [30]. Expression of DNMIκBα significantly inhibited the *Mcl-1* promoter activity in TE-1 and KYSE150 cells (Figure 3A). Furthermore, compared with their respective DMSO control, treatment with 20 μM Bay11-7082, a specific NF-κB inhibitor, resulted in the *Mcl-1* promoter activity drastically curtailed in both TE-1 and KYSE150 cells. The activity of the *Mcl-1* promoter with mutated NF-κB site was essentially unaffected by inhibitor treatment (Figure 3A). NF-κB transcriptional activities in both TE-1 and KYSE150 cell lines have also been estimated by using an NF-κB-driven luciferase reporter. The results indicated that NF-κB-driven luciferase reporter show an increased transcriptional activity in both TE-1 and KYSE150 cells compared with the vector control (Figure 3B). Bay11-7082 (20 μM) significantly attenuated the increased transcriptional activity of NF-κB-driven luciferase reporter in these two cell lines, thus confirmed the efficiency of Bay11-7082 as an NF-κB inhibitor (Figure 3B). Notably, the increased transcriptional activity of the *Mcl-1* promoter observed in Eca109 cells remained unchanged by the above three strategies (Figure 3A). Taken together, these results provide consistent evidence that the involvement of NF-κB pathway in the *Mcl-1* promoter transcriptional activity in various human ESCC cells.

### NF-κB signaling pathway contributes to Mcl-1 expression in various human esophageal squamous cell carcinoma cell lines

**Figure 4:**
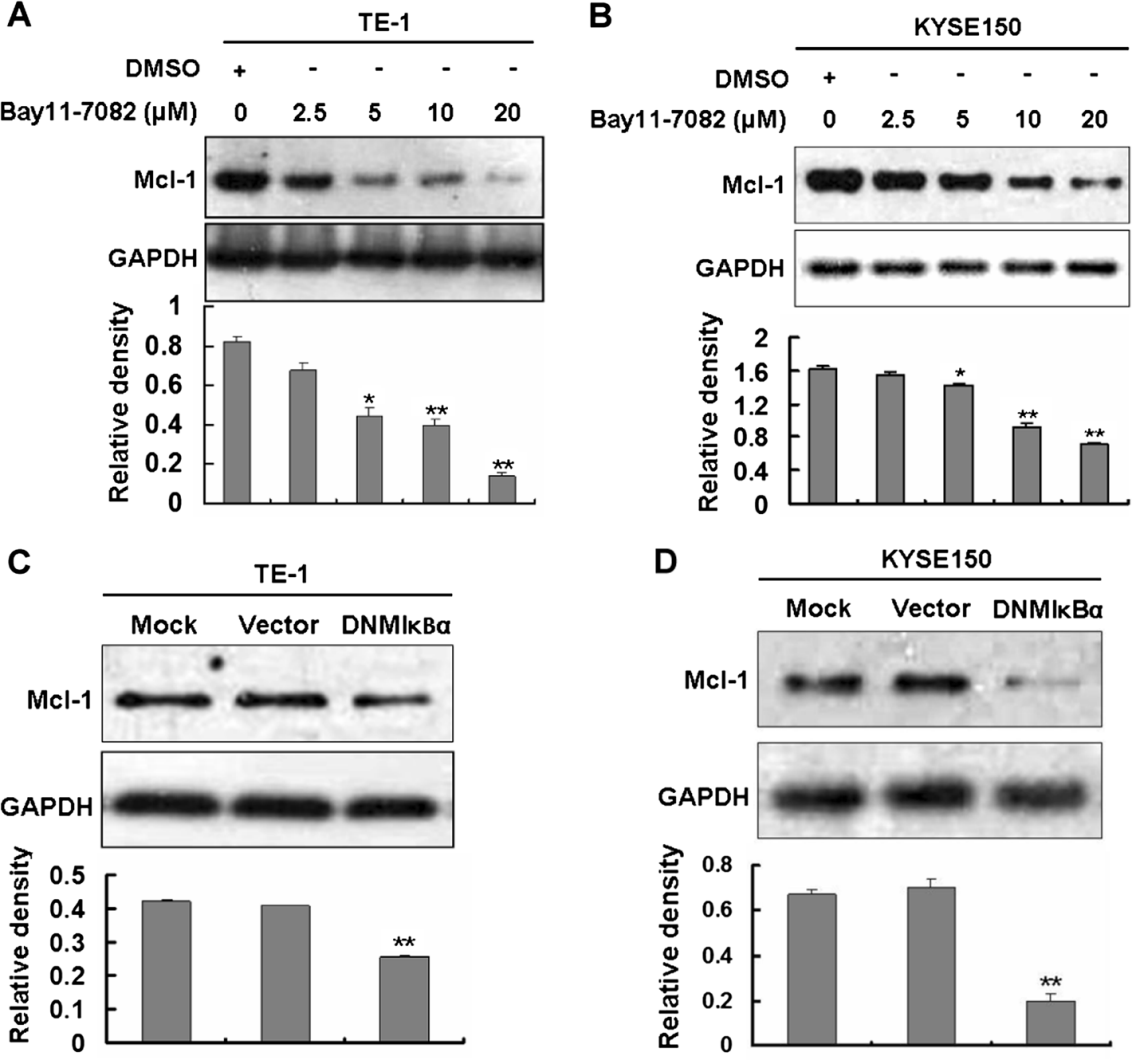
**Attenuation of Mcl-1 expression by NF-κB inhibitor or dominant negative mutant of IκBα in various human esophageal squamous cell carcinoma cell lines. (A, B)** Inhibition of NF-κB pathway by NF-κB-specific inhibitor Bay11-7082 prevented Mcl-1 expression in TE-1 **(A)** and KYSE150 **(B)** cell lines. TE-1 or KYSE150 cells were treated with indicated concentrations of DMSO or Bay11-7082 for 24 h, whole cell lysates were harvested. Mcl-1 was determined by Western blotting. GAPDH was used as a loading control. Data shown are representative of at least two independent experiments. Statistical significance: * p < 0.05 and **p < 0.01, compared with the DMSO control. **(C, D)** Expression of dominant negative mutant of IκBα (DNMIκBα) decreased Mcl-1 protein level in TE-1 **(C)** and KYSE150 **(D)** cell lines. TE-1 or KYSE150 cells seeded in 12-well plate were untransfected (Mock), transfected with 1600 ng/well pcDNA3.1 empty vector or 1600 ng/well DNMIκBα expression plasmid using Lipofectamine™ 2000 according to manufacturer’s instructions. Cells were harvested at 24 h after transfection and subjected to Western blotting analysis with anti-Mcl-1 antibody. GAPDH was used as a loading control. Data shown are representative of at least two independent experiments. Statistical significance: **p < 0.01, compared with the pcDNA3.1 empty vector-transfected control.

We further confirm whether NF-κB is involved in Mcl-1 expression in human ESCC cells. Bay11-7082 was firstly used to investigate the effect of NF-κB activation on Mcl-1 induction. Treatment of TE-1 cells with the inhibitor resulted in a dose-dependent attenuation of Mcl-1 induction (Figure 4A). Similar results were obtained from KYSE150 cells treated with various concentrations of Bay11-7082 (Figure 4B). DNMIκBα was further used to test the role of NF-κB pathway in regulating Mcl-1 expression. As verified by Western blotting analysis, expression of DNMIκBα in TE-1 (Figure 4C) or KYSE150 (Figure 4D) cells led to a significant decrease of Mcl-1 induction compared with the vector control. The results suggested that NF-κB pathway is involved in Mcl-1 expression in TE-1 and KYSE150 cells.

### Binding of transcription factor NF-κB family members to human *Mcl-1* promoter

**Figure 5:**
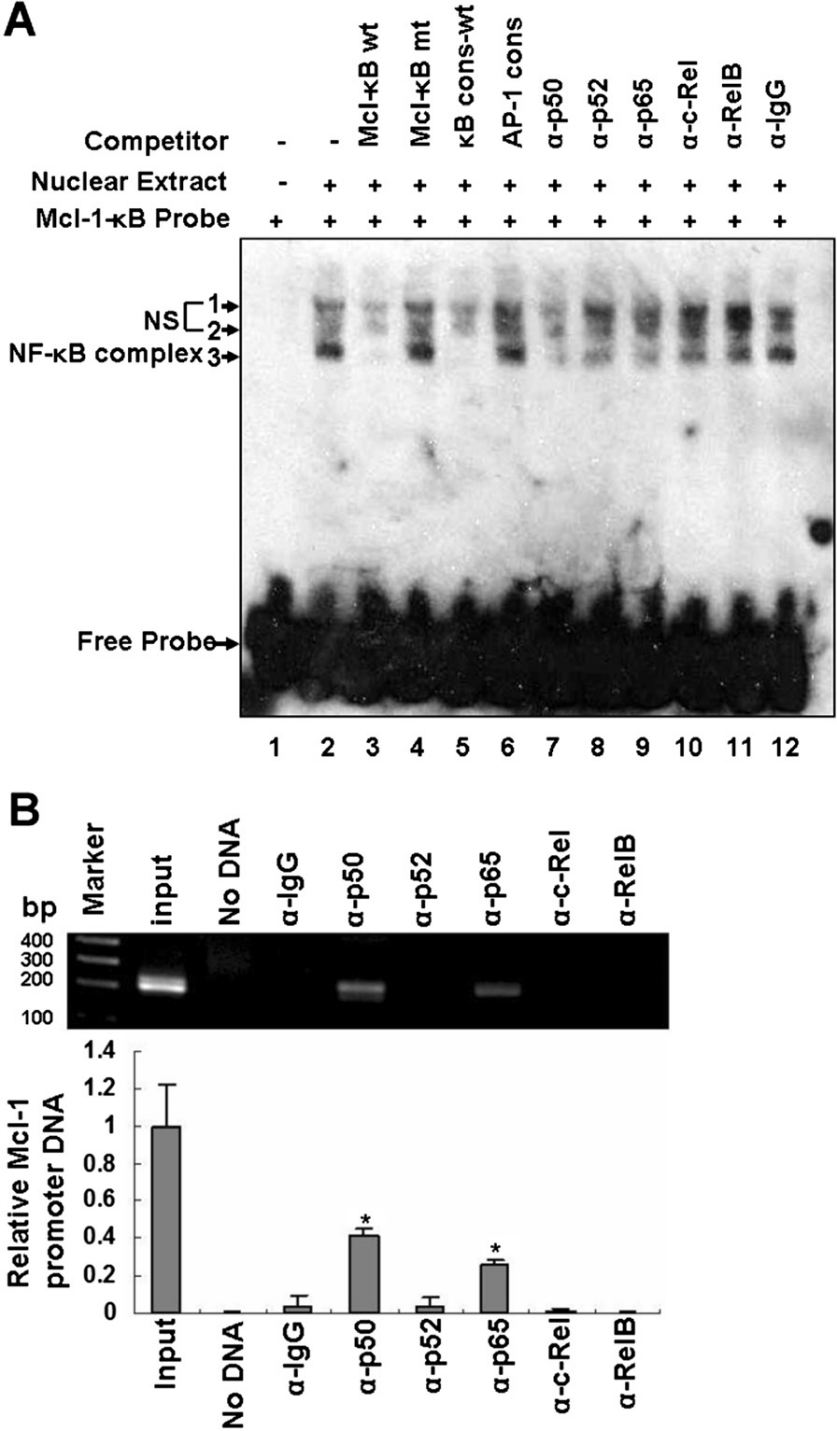
**Binding of transcription factor NF-κB to human *Mcl-1 *promoter. (A)** Binding of NF-κB transcription factor to Mcl-1-κB probe *in vitro.* Nuclear extracts of TE-1 cells were prepared for EMSA. Nuclear extracts were preincubated with biotin-labeled Mcl-1-κB oligonucleotide probe in the absence or presence of antibodies directed against different NF-κB subunits p50, p52, p65, c-Rel, RelB or control antibody (IgG) (indicated above each lane) and then gel shift assays were performed. The arrows indicated the DNA-protein complexes. Free labeled probes are also indicated. The results are representative of two independent experiments. NS, nonspecific band. **(B)** Transcription factor NF-κB p50 and p65 subunits directly interacted with human *Mcl-1* promoter in intact cells. The cross-linked chromatin was precipitated with antibody against transcription factor NF-κB subunit p50, p52, p65, c-Rel or RelB. The positive control is represented by the input fraction. Negative controls included a no chromatin sample and a nonspecific antibody (α-IgG) sample. Precipitated DNA was analyzed by PCR using primers that amplified a 200-bp region, which included the NF-κB binding site of human *Mcl-1* promoter. Data represented the mean ± S.D. of two separate experiments. Statistical significance: * p < 0.05, compared with the IgG control.

To ascertain whether NF-κB transcription factor can bind the NF-κB site in human *Mcl-1* promoter, EMSA was performed with an oligonucleotide probe containing the putative NF-κB binding sequence derived from human *Mcl-1* promoter. Three DNA-protein complexes were evident with nuclear extracts from TE-1 cells, labeled bands 1, 2 and 3, respectively (Figure 5A). To further confirm whether these three bands are specific for the NF-κB complexes, a competition assay was performed. The band 3 of complex could be completely abolished by a 100-fold excess unlabeled wild-type Mcl-1-κB probe (lane 3) or NF-κB consensus oligonucleotide (lane 5), but not by 100-fold excess unlabeled mutant Mcl-1*-*κB probe (lane 4) or 100-fold excess unrelated AP-1 consensus oligonucleotide (lane 6). In contrast, two upper bands (1 and 2) were not competed away by either unlabeled wild-type Mcl-1*-*κB oligonucleotide (lane 3) or κB consensus probe (lane 5) even at a 100-fold molar excess. These results, which were similar to previously published report [39], suggested that the band 3 is specific for the NF-κB complex. The observation that the Mcl-1*-*κB oligonucleotide can bind non-NF-κB specific complexes as well might due to other protein(s) present in the nuclear extracts that also bind the NF-κB sequence of the oligonucleotide [40]. To identify which components of NF-κB contribute to this binding activity, supershift analysis was performed with nuclear extracts from TE-1 cells. In the presence of antibodies against NF-κB subunits p50, p52, p65, c-Rel, and RelB, the results revealed that the addition of an antibody against p50, p52 or p65 caused a substantial reduction in binding (lanes 7, 8 and 9). The intensity of the DNA-protein complex was slightly depleted by c-Rel (lane 10) while antibody against RelB had no effect on binding (lane 11). IgG control also showed no effect on the intensity of the complex (lane 12). These data demonstrated that binding of these antibodies prevents association with the labeled probe. The decreases in band intensity suggested the presence of these transcription factors in the complex, which indicate that p50, p52 and p65 are the major NF-κB subunits binding to the human Mcl-1-κB probe *in vitro*.

To determine whether transcription factor NF-κB actually bind to human *Mcl-1* promoter in intact cells, we analyzed the fragment that spans the NF-κB binding region within human *Mcl-1* promoter using a chromatin immunoprecipitation assay (ChIP). The sheared cross-linked chromatin of TE-1 cells was immunoprecipitated by antibodies specific for NF-κB subunits p50, p52, p65, c-Rel and RelB. An IgG antibody was used as a nonspecific control. The precipitated chromatin DNA was then amplified by PCR using primers specific for NF-κB binding site of human *Mcl-1* gene, which produced 200-bp amplicons that could be observed with the positive control (input chromatin) and when the chromatin was precipitated with antibodies for p50 and p65, respectively. No amplification was observed with two negative controls (no chromatin and IgG) (Figure 5B). The ChIP results indicated that NF-κB subunits p50 and p65 can exert their regulatory function through directly binding to the NF-κB site of human *Mcl-1* promoter and finally regulating Mcl-1 expression in TE-1 cells. Overall, the results suggested that the interaction of transcription factor NF-κB subunits p50 and p65 with human *Mcl-1* promoter might be a key event in the regulation of Mcl-1 expression in TE-1 cells.

### Knockdown of NF-κB subunit attenuates Mcl-1 expression and inhibits TE-1 cell viability

**Figure 6:**
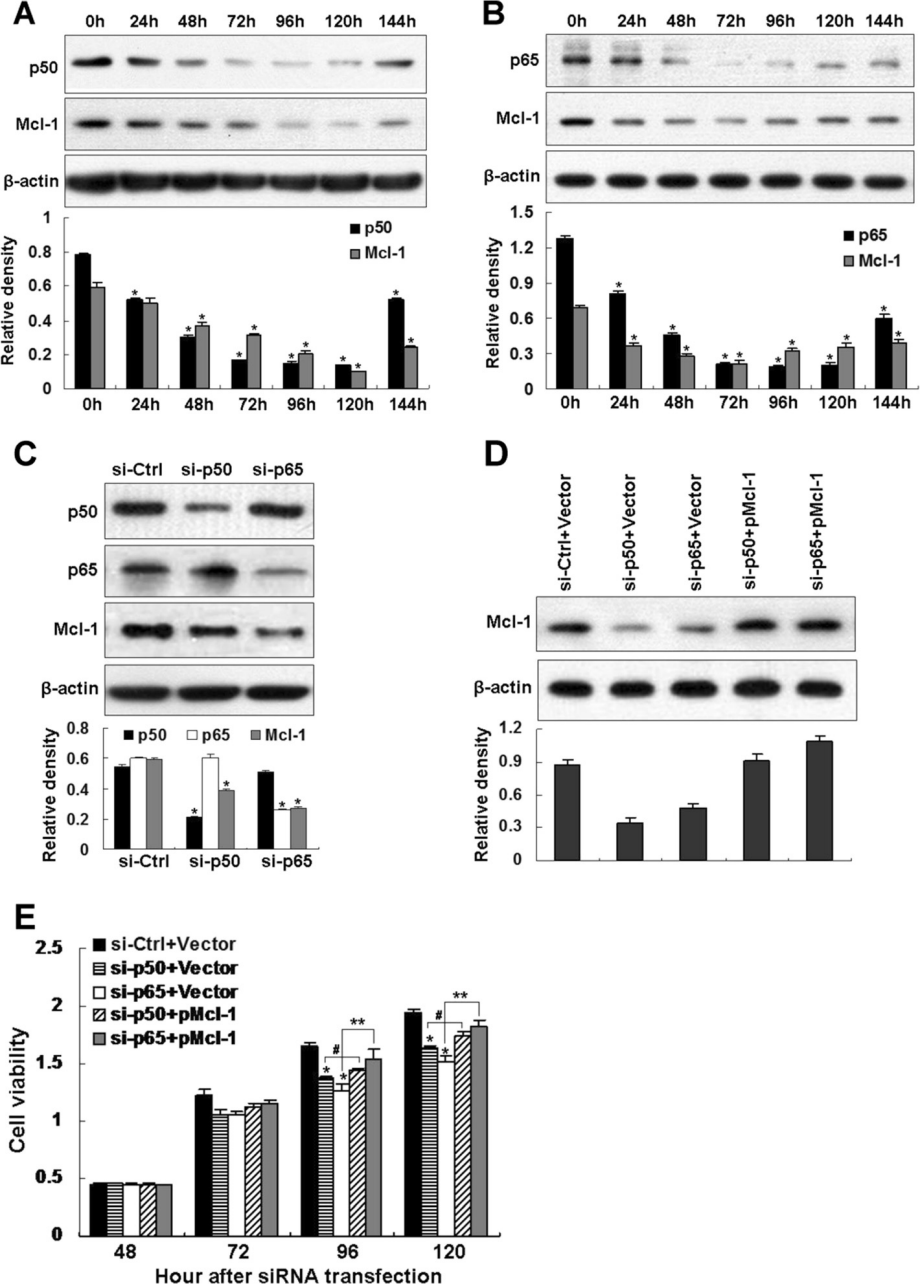
**Inhibition of NF-κB subunit by siRNA downregulates Mcl-1 expression and suppresses the viability of TE-1 cells. (A, B)** Time course experiments performed with p50 or p65 siRNA utilized. Representative Western blots of endogenous p50, p65 or Mcl-1expression in TE-1 cells at various time points following transfection with 100 nM p50 **(A)** or p65 **(B)** siRNA. β-actin was used as a loading control. Data shown are representative of two independent experiments. Statistical significance: * p < 0.05, compared with the siRNA-transfected TE-1 cells at 0 h. **(C)** Growing TE-1 cells were transiently transfected with 100 nM of control, p50 or p65 siRNA and cultured in the medium for 72 h. Knockdown of endogenous p50 or p65 and expression of Mcl-1 were analyzed by Western blotting. β-actin was used as a loading control. Data represented the mean ± S.D. of two separate experiments. Statistical significance: * p < 0.05 compared with the si-Ctrl-transfected TE-1 cells. **(D, E)** Reintroduction of Mcl-1 to Mcl-1-downregulated TE-1 cells caused by NF-κB subunit siRNA restored cell viability. TE-1 cells were sequentially transfected with NF-κB subunit siRNA and Mcl-1 expression plasmid as described in Methods. Cells were analyzed the Mcl-1 levels at 120 h after siRNA transfection by Western blotting **(D)** and measured cell viability by WST-1 assay at 24 h-intervals up to 120 h after siRNA transfection **(E)**. Data represented the mean ± S.D. of two separate experiments. Statistical significance: * p < 0.05, compared with the si-Ctrl and pCMV6-A-Puro empty vector co-transfected TE-1 cells. # p < 0.05, compared the si-p50 and pCMV6-A-Puro empty vector co-transfected with the si-p50 and pCMV6-A-Puro-Mcl co-transfected TE-1 cells. ** p < 0.05, compared the si-p65 and pCMV6-A-Puro empty vector co-transfected with the si-p65 and pCMV6-A-Puro-Mcl co-transfected TE-1 cells.

To further confirm the involvement of individual NF-κB subunits in Mcl-1 expression, we performed knockdown experiments. TE-1 cells were transfected with siRNAs to either p50, p65 or a scrambled control and then the Mcl-1 levels were assessed. To determine the optimal time point for analysis, a time-course experiment was performed at multiple time points after transfection. Representative time-course data of Mcl-1 reduced by p50 or p65 siRNA was shown in Figure 6A and B. The levels of endogenous p50 and p65 decreased by 24 h after transfection of si-p50 or si-p65 and peaked 72 h, then gradually recovered with time. The Mcl-1 downregulation peaked 96 h after si-p50 transfection (Figure 6A) and peaked 72 h after si-p65 transfection (Figure 6B) and remained at relatively low levels 144 h posttransfection. Base on the time-course data, the optimal protocol of 72 h-treatment was used in subsequent experiments. Compared with the control siRNA, silencing of p50 or p65 each simultaneously led to a significant decrease of Mcl-1 protein levels (Figure 6C). With these data confirming the knockdown of NF-κB subunits and the downregulation of Mcl-1 expression, we next tested the effect of the NF-κB subunit siRNAs on TE-1 cell viability. Silencing of p50 or p65 resulted in decrease of Mcl-1 level (Figure 6D), which significantly inhibited the viability of TE-1 cells (Figure 6E). Reintroduction of human Mcl-1 (Figure 6D) significantly restored cell viability (Figure 6E), indicating that the specific reduction of Mcl-1 by p50 or p65 siRNA. Notably, cell viability was unable to be completely rescued even the Mcl-1 levels were totally recovered, suggesting other NF-κB-dependent proteins might also contribute to TE-1 cell viability. These results suggest that NF-κB subtypes formed functional heterodimers mediating Mcl-1 expression and cell viability in TE-1 cells.

## Discussion

Expression of Mcl-1 is frequently increased in various human tumors, so the mechanisms that increase Mcl-1 levels are of paramount importance. In addition to being modulated at transcriptional level by various transcription factors that bind and activate the *Mcl-1* promoter aforementioned, Mcl-1 could be regulated on multiple levels, such as translational and post-translational. For instance, E3 ubiquitin ligase Mule has been identified to required and sufficient for the polyubiquitination of Mcl-1. Elimination of Mule expression by RNA interference stabilizes Mcl-1 protein, resulting in an increase of Mcl-1 protein level [41]. Another E3 ligase β-TrCP facilitates the ubiquitination and degradation of GSK-3β-phosphorylated Mcl-1, which contributes to GSK-3β-induced apoptosis [42]. Mutational inactivation of E3 ligase FBW7 was found to occur in several neoplastic diseases, which can decrease Mcl-1 degradation, resulting in increased Mcl-1 protein levels and resistance to chemotherapeutic agents [43]. In contrast, deubiquitinase USP9X, which is overexpressed in some malignancies, stabilizes Mcl-1 and promotes tumor cell survival. Knockdown of USP9X decreased Mcl-1 levels [5]. Moreover, phosphorylation of Mcl-1 at Thr 163 by ERK [43] prolongs the Mcl-1 half-life while phosphorylation at Thr 163 by GSK-3β [42] or Thr 92 by CDK1 [43] enhances Mcl-1 degradation. In addition, Mcl-1 transcripts can be influenced by microRNAs (miRs). For example, miR29b has been demonstrated to downregulate Mcl-1 protein and sensitize cells to apoptosis [44]. Future studies need to explore whether these mechanisms contribute to the elevated Mcl-1 protein in human ESCC.

Increased Mcl-1 protein level has been reported to compromise the apoptotic effects of chemotherapeutic agents, resulting in therapeutic resistance [43]. Thus, the pathways that are critical for regulating Mcl-1 expression have been employed to target Mcl-1 for cancer therapy. For instance, in large granular lymphocyte leukemia, targeting Stat3 with its upstream kinase JAK-selective inhibitor AG490 transcriptionally suppresses Mcl-1 and promotes apoptosis [12]. PI3K/Akt signaling is involved in Mcl-1 induction [10], targeting this pathway by newly developed PI3K inhibitor PI103 is showed to suppress Mcl-1 and induced apoptosis and restore sensitivity to TRAIL-induced apoptosis in neuroblastoma [45]. Treatment with MEK/ERK inhibitor U0126 resulted in Mcl-1 downregulation and induced marked apoptosis in Mel-RM melanoma cells [46]. Therefore, identification of pathways that regulate Mcl-1 may help to improve the therapeutic effect of chemotherapy. Our data indicated that inhibition of NF-κB pathway by Bay11-7082 (Figure 4A, B), DNMIκBα (Figure 4C, D) or NF-κB subunit siRNA (Figure 6) attenuates Mcl-1 expression in human ESCC cells. We also found that the survival of TE-1 cells is impaired when NF-κB is blocked by expression of p50 siRNA or p65 siRNA and reintroduction of Mcl-1 to the siRNA-transfected TE-1 cells significantly restores cell viability (Figure 6E). These data that decrease Mcl-1 expression and inhibits cell viability by inhibition of NF-κB pathway support the use of selective NF-κB inhibitors in the treatment of Mcl-1-overexpressing human ESCC.

By gel shift analysis, nuclear extracts of TE-1 cells were preincubated with antisera directed against individual NF-κB family members p50, p52, p65, c-Rel, RelB or with a nonspecific antisera prior to interaction with the Mcl-1-κB site probe. We found that NF-κB family members p50, p52 and p65 were able to bind to the same probe *in vitro*. The result was in agreement with the earlier findings that most κB sites show no or little selectivity for a given NF-κB species and different dimers have broad sequence recognition specificities although relatively small differences in the relative affinity of NF-κB dimers for a given site can be found [47-49]. However, p50 and p65 but not p52 were revealed directly binding to the κB site of human *Mcl-1* promoter in intact cells by ChIP assays. The discrepancy between the measured *in vitro* affinity of NF-κB for the κB probe and the real *in vivo* occupancy at κB site of the natural promoter is not without precedent. For instance, ChIP result showed that, in LPS-stimulated DCs, the κB site of *IL-8* promoter is a highly selective p65 recruiter [50], while in *in vitro* experiments, it is bound and activated by both p65 and c-Rel homodimers [51]. The ability of a specific gene to selectively recruit various NF-κB dimers in vivo cannot be predicted on the basis of in vitro results [50]. The context of κB site physiological promoter rather than the κB site itself is the major determinant of which NF-κB dimmer will ultimately be loaded onto a certain promoter.

Although putative binding sites for NF-κB were identified in the *Mcl-1* promoter region [9] and two recent reports have shown that NF-κB is directly involved in Mcl-1 regulation [17,18]. In the first article, by using ChIP assay, the authors show that p65 subunit of NF-κB following TRAIL treatment binds to the *Mcl-1* promoter, which suggested that TRAIL induced expression of Mcl-1 through activation of NF-κB in HCT-116 colon carcinoma cells [17]. In the second study, the authors show that transcriptional activation of *Mcl-1* gene required the recruitment of N-a-Acetyltransferase 10 protein/p65 complex to the p65-binding site of the *Mcl-1* promoter region [18]. However, both studies focused only on the role of NF-κB p65 subunit in Mcl-1 expression and the report of other NF-κB subunits involved in Mcl-1 expression is relatively limited. Since dimerization is required for NF-κB binding to DNA and more than 12 homo- and heterodimers have been described [50]. The analysis of other members of the NF-κB family to bind to κB site and regulate Mcl-1 expression would allow for a better understanding of the precise mechanism of *Mcl-1* transcriptional control by NF-κB. Our results indicate that effect of NF-κB on Mcl-1 expression in TE-1 cells is due to activation of NF-κB subtypes p65 and p50, without activation of other subtypes (Figure 5B) and reveal that activations of p65 and p50 are involved in Mcl-1 expression thus affecting cell viability (Figure 6E). Notably, we did not observe the involvement of NF-κB pathway in human *Mcl-1* promoter activity in Eca109 cells (Figure 3A). In addition to NF-κB binding site, the 325 bp long *Mcl-1* promoter fragment contains CRE-BP, Ets, Sp1, SRE, STAT binding sites [17,52]. We speculated that, in Eca109 cells, other transcription factor(s) rather than NF-κB might play a leading role in Mcl-1 expression. Our results suggested that the existence of other regulatory cascades that modulate Mcl-1 expression in different ESCC cells.

## Conclusions

In summary, we provided evidence regarding how *Mcl-1* is regulated at transcription level in human ESCC cell lines. The present study demonstrated that NF-κB contributes to Mcl-1 production in various human ESCC cells and subunits p50 and p65 of NF-κB positively regulate Mcl-1 expression and cell viability in TE-1 cells. The results support the conclusion that Mcl-1 plays a key role in mediating TE-1 cell fate downstream of the NF-κB pathway. The newly identified mechanism suggests that targeting the NF-κB pathway might improve treatment results in some human ESCCs with high Mcl-1 expression.

## Competing interests

The authors declare that they have no competing interests.

## Authors’ contributions

HDL conceived the study, analyzed data and drafted the manuscript. JFY YCY acquired and analyzed data. ZKX MJC JW LX XLM acquired data. SFO QW provided material support. XMZ YFY FLY YC reviewed the manuscript. BLY JGH supervised the study, analyzed data and finalized the manuscript. All authors read and approved the final manuscript.

## Pre-publication history

The pre-publication history for this paper can be accessed here:

http://www.biomedcentral.com/1471-2407/14/98/prepub

## References

[B1] Enzinger PC Mayer RJ Esophageal cancer N Engl J Med 2003 349 2241 2252 10.1056/NEJMra035010 14657432

[B2] Sieghart W Losert D Strommer S Cejka D Schmid K Rasoul-Rockenschaub S Bodingbauer M Crevenna R Monia BP Peck-Radosavljevic M Wacheck V Mcl-1 overexpression in hepatocellular carcinoma: a potential target for antisense therapy J Hepatol 2006 44 151 157 10.1016/j.jhep.2005.09.010 16289418

[B3] Miyamoto Y Hosotani R Wada M Lee JU Koshiba T Fujimoto K Tsuji S Nakajima S Doi R Kato M Immunohistochemical analysis of Bcl-2, Bax, Bcl-X, and Mcl-1 expression in pancreatic cancers Oncology 1999 56 73 82 10.1159/000011933 9885381

[B4] Dash R Richards JE Su ZZ Bhutia SK Azab B Rahmani M Dasmahapatra G Yacoub A Dent P Dmitriev IP Mechanism by which Mcl-1 regulates cancer-specific apoptosis triggered by mda-7/IL-24, an IL-10-related cytokine Cancer Res 2010 70 5034 5045 10.1158/0008-5472.CAN-10-0563 20501829 PMC3171699

[B5] Schwickart M Huang X Lill JR Liu J Ferrando R French DM Maecker H O’Rourke K Bazan F Eastham-Anderson J Deubiquitinase USP9X stabilizes MCL1 and promotes tumour cell survival Nature 2010 463 103 107 10.1038/nature08646 20023629

[B6] Keuling AM Felton KE Parker AA Akbari M Andrew SE Tron VA RNA silencing of Mcl-1 enhances ABT-737-mediated apoptosis in melanoma: role for a caspase-8-dependent pathway PLoS One 2009 4 e6651 10.1371/journal.pone.0006651 19684859 PMC2722728

[B7] Beroukhim R Mermel CH Porter D Wei G Raychaudhuri S Donovan J Barretina J Boehm JS Dobson J Urashima M The landscape of somatic copy-number alteration across human cancers Nature 2010 463 899 905 10.1038/nature08822 20164920 PMC2826709

[B8] Placzek WJ Wei J Kitada S Zhai D Reed JC Pellecchia M A survey of the anti-apoptotic Bcl-2 subfamily expression in cancer types provides a platform to predict the efficacy of Bcl-2 antagonists in cancer therapy Cell Death Dis 2010 1 e40 10.1038/cddis.2010.18 21364647 PMC3032312

[B9] Akgul C Turner PC White MR Edwards SW Functional analysis of the human MCL-1 gene Cell Mol Life Sci 2000 57 684 691 10.1007/PL00000728 11130466 PMC11147037

[B10] Wang JM Chao JR Chen W Kuo ML Yen JJ Yang-Yen HF The antiapoptotic gene mcl-1 is up-regulated by the phosphatidylinositol 3-kinase/Akt signaling pathway through a transcription factor complex containing CREB Mol Cell Biol 1999 19 6195 6206 10454566 10.1128/mcb.19.9.6195 PMC84561

[B11] Liu H Ma Y Cole SM Zander C Chen KH Karras J Pope RM Serine phosphorylation of STAT3 is essential for Mcl-1 expression and macrophage survival Blood 2003 102 344 352 10.1182/blood-2002-11-3396 12637318

[B12] Epling-Burnette PK Liu JH Catlett-Falcone R Turkson J Oshiro M Kothapalli R Li Y Wang JM Yang-Yen HF Karras J Inhibition of STAT3 signaling leads to apoptosis of leukemic large granular lymphocytes and decreased Mcl-1 expression J Clin Invest 2001 107 351 362 10.1172/JCI9940 11160159 PMC199188

[B13] Townsend KJ Zhou P Qian L Bieszczad CK Lowrey CH Yen A Craig RW Regulation of MCL1 through a serum response factor/Elk-1-mediated mechanism links expression of a viability-promoting member of the BCL2 family to the induction of hematopoietic cell differentiation J Biol Chem 1999 274 1801 1813 10.1074/jbc.274.3.1801 9880563

[B14] Wang JM Lai MZ Yang-Yen HF Interleukin-3 stimulation of mcl-1 gene transcription involves activation of the PU.1 transcription factor through a p38 mitogen-activated protein kinase-dependent pathway Mol Cell Biol 2003 23 1896 1909 10.1128/MCB.23.6.1896-1909.2003 12612065 PMC149468

[B15] Meinel FG Mandl-Weber S Baumann P Leban J Schmidmaier R The novel, proteasome-independent NF-kappaB inhibitor V1810 induces apoptosis and cell cycle arrest in multiple myeloma and overcomes NF-kappaB-mediated drug resistance Mol Cancer Ther 2010 9 300 310 20124446 10.1158/1535-7163.MCT-09-0645

[B16] Reuter S Prasad S Phromnoi K Ravindran J Sung B Yadav VR Kannappan R Chaturvedi MM Aggarwal BB Thiocolchicoside exhibits anticancer effects through downregulation of NF-kappaB pathway and its regulated gene products linked to inflammation and cancer Cancer Prev Res (Phila) 2010 3 1462 1472 10.1158/1940-6207.CAPR-10-0037 20978115 PMC3142676

[B17] Ricci MS Kim SH Ogi K Plastaras JP Ling J Wang W Jin Z Liu YY Dicker DT Chiao PJ Reduction of TRAIL-induced Mcl-1 and cIAP2 by c-Myc or sorafenib sensitizes resistant human cancer cells to TRAIL-induced death Cancer Cell 2007 12 66 80 10.1016/j.ccr.2007.05.006 17613437

[B18] Xu H Jiang B Meng L Ren T Zeng Y Wu J Qu L Shou C N-alpha-acetyltransferase 10 protein inhibits apoptosis through RelA/p65-regulated MCL1 expression Carcinogenesis 2012 33 1193 1202 10.1093/carcin/bgs144 22496479

[B19] Perkins ND Integrating cell-signalling pathways with NF-kappaB and IKK function Nat Rev Mol Cell Biol 2007 8 49 62 10.1038/nrm2083 17183360

[B20] Perkins ND Regulation of NF-kappaB by atypical activators and tumour suppressors Biochem Soc Trans 2004 32 936 939 15506929 10.1042/BST0320936

[B21] Sethi G Sung B Aggarwal BB Nuclear factor-kappaB activation: from bench to bedside Exp Biol Med (Maywood) 2008 233 21 31 10.3181/0707-MR-196 18156302

[B22] Basseres DS Baldwin AS Nuclear factor-kappaB and inhibitor of kappaB kinase pathways in oncogenic initiation and progression Oncogene 2006 25 6817 6830 10.1038/sj.onc.1209942 17072330

[B23] Izzo JG Malhotra U Wu TT Ensor J Luthra R Lee JH Swisher SG Liao Z Chao KS Hittelman WN Association of activated transcription factor nuclear factor kappab with chemoradiation resistance and poor outcome in esophageal carcinoma J Clin Oncol 2006 24 748 754 10.1200/JCO.2005.03.8810 16401681

[B24] Tian F Zang WD Hou WH Liu HT Xue LX Nuclear factor-kB signaling pathway constitutively activated in esophageal squamous cell carcinoma cell lines and inhibition of growth of cells by small interfering RNA Acta Biochim Biophys Sin (Shanghai) 2006 38 318 326 10.1111/j.1745-7270.2006.00166.x 16680372

[B25] Leu CM Chang C Hu C Epidermal growth factor (EGF) suppresses staurosporine-induced apoptosis by inducing mcl-1 via the mitogen-activated protein kinase pathway Oncogene 2000 19 1665 1675 10.1038/sj.onc.1203452 10763823

[B26] Feng YB Lin DC Shi ZZ Wang XC Shen XM Zhang Y Du XL Luo ML Xu X Han YL Overexpression of PLK1 is associated with poor survival by inhibiting apoptosis via enhancement of survivin level in esophageal squamous cell carcinoma Int J Cancer 2009 124 578 588 10.1002/ijc.23990 19004025

[B27] Boukamp P Petrussevska RT Breitkreutz D Hornung J Markham A Fusenig NE Normal keratinization in a spontaneously immortalized aneuploid human keratinocyte cell line J Cell Biol 1988 106 761 771 10.1083/jcb.106.3.761 2450098 PMC2115116

[B28] Yang LF Zhao Y Zeng L Gong JP Cao Y hTERT/re-caspase-3 system induce apoptosis in hTERT-positive cancer cells Cancer Biol Ther 2006 5 1546 1553 10.4161/cbt.5.11.3460 17172814

[B29] Liu HD Zheng H Li M Hu DS Tang M Cao Y Upregulated expression of kappa light chain by Epstein-Barr virus encoded latent membrane protein 1 in nasopharyngeal carcinoma cells via NF-kappaB and AP-1 pathways Cell Signal 2007 19 419 427 10.1016/j.cellsig.2006.07.012 16979873

[B30] Liu H Zheng H Duan Z Hu D Li M Liu S Li Z Deng X Wang Z Tang M LMP1-augmented kappa intron enhancer activity contributes to upregulation expression of Ig kappa light chain via NF-kappaB and AP-1 pathways in nasopharyngeal carcinoma cells Mol Cancer 2009 8 92 10.1186/1476-4598-8-92 19860880 PMC2774294

[B31] Luo W Yan G Li L Wang Z Liu H Zhou S Liu S Tang M Yi W Dong Z Cao Y Epstein-Barr virus latent membrane protein 1 mediates serine 25 phosphorylation and nuclear entry of annexin A2 via PI-PLC-PKCalpha/PKCbeta pathway Mol Carcinog 2008 47 934 946 10.1002/mc.20445 18412141

[B32] Liu H Duan Z Zheng H Hu D Li M Tao Y Bode AM Dong Z Cao Y EBV-encoded LMP1 upregulates Igkappa 3′enhancer activity and Igkappa expression in nasopharyngeal cancer cells by activating the Ets-1 through ERKs signaling PLoS One 2012 7 e32624 10.1371/journal.pone.0032624 22396784 PMC3291551

[B33] Rosato RR Almenara JA Kolla SS Maggio SC Coe S Gimenez MS Dent P Grant S Mechanism and functional role of XIAP and Mcl-1 down-regulation in flavopiridol/vorinostat antileukemic interactions Mol Cancer Ther 2007 6 692 702 10.1158/1535-7163.MCT-06-0562 17308065

[B34] Solanas G Porta-de-la-Riva M Agusti C Casagolda D Sanchez-Aguilera F Larriba MJ Pons F Peiro S Escriva M Munoz A E-cadherin controls beta-catenin and NF-kappaB transcriptional activity in mesenchymal gene expression J Cell Sci 2008 121 2224 2234 10.1242/jcs.021667 18565826

[B35] Wang X Sonenshein GE Induction of the RelB NF-kappaB subunit by the cytomegalovirus IE1 protein is mediated via Jun kinase and c-Jun/Fra-2 AP-1 complexes J Virol 2005 79 95 105 10.1128/JVI.79.1.95-105.2005 15596805 PMC538727

[B36] Sohda M Mochida Y Kato H Miyazaki T Nakajima M Fukuchi M Manda R Fukai Y Masuda N Ono M Overexpression of Cap43 is associated with malignant status of esophageal cancer Anticancer Res 2009 29 965 970 19414333

[B37] Yan S Zhou C Zhang W Zhang G Zhao X Yang S Wang Y Lu N Zhu H Xu N beta-Catenin/TCF pathway upregulates STAT3 expression in human esophageal squamous cell carcinoma Cancer Lett 2008 271 85 97 10.1016/j.canlet.2008.05.035 18602747

[B38] Li Y Reddy MA Miao F Shanmugam N Yee JK Hawkins D Ren B Natarajan R Role of the histone H3 lysine 4 methyltransferase, SET7/9, in the regulation of NF-kappaB-dependent inflammatory genes. Relevance to diabetes and inflammation J Biol Chem 2008 283 26771 26781 10.1074/jbc.M802800200 18650421 PMC2546554

[B39] Huang C Bi E Hu Y Deng W Tian Z Dong C Hu Y Sun B A novel NF-kappaB binding site controls human granzyme B gene transcription J Immunol 2006 176 4173 4181 16547254 10.4049/jimmunol.176.7.4173

[B40] Beverly LJ Capobianco AJ Targeting promiscuous signaling pathways in cancer: another Notch in the bedpost Trends Mol Med 2004 10 591 598 10.1016/j.molmed.2004.10.001 15567329

[B41] Zhong Q Gao W Du F Wang X Mule/ARF-BP1, a BH3-only E3 ubiquitin ligase, catalyzes the polyubiquitination of Mcl-1 and regulates apoptosis Cell 2005 121 1085 1095 10.1016/j.cell.2005.06.009 15989957

[B42] Ding Q He X Hsu JM Xia W Chen CT Li LY Lee DF Liu JC Zhong Q Wang X Hung MC Degradation of Mcl-1 by beta-TrCP mediates glycogen synthase kinase 3-induced tumor suppression and chemosensitization Mol Cell Biol 2007 27 4006 4017 10.1128/MCB.00620-06 17387146 PMC1900029

[B43] Wertz IE Kusam S Lam C Okamoto T Sandoval W Anderson DJ Helgason E Ernst JA Eby M Liu J Sensitivity to antitubulin chemotherapeutics is regulated by MCL1 and FBW7 Nature 2011 471 110 114 10.1038/nature09779 21368834

[B44] Mott JL Kobayashi S Bronk SF Gores GJ mir-29 regulates Mcl-1 protein expression and apoptosis Oncogene 2007 26 6133 6140 10.1038/sj.onc.1210436 17404574 PMC2432524

[B45] Opel D Naumann I Schneider M Bertele D Debatin KM Fulda S Targeting aberrant PI3K/Akt activation by PI103 restores sensitivity to TRAIL-induced apoptosis in neuroblastoma Clin Cancer Res 2011 17 3233 3247 10.1158/1078-0432.CCR-10-2530 21355080

[B46] Wang YF Jiang CC Kiejda KA Gillespie S Zhang XD Hersey P Apoptosis induction in human melanoma cells by inhibition of MEK is caspase-independent and mediated by the Bcl-2 family members PUMA, Bim, and Mcl-1 Clin Cancer Res 2007 13 4934 4942 10.1158/1078-0432.CCR-07-0665 17652623

[B47] Udalova IA Mott R Field D Kwiatkowski D Quantitative prediction of NF-kappa B DNA-protein interactions Proc Natl Acad Sci U S A 2002 99 8167 8172 10.1073/pnas.102674699 12048232 PMC123039

[B48] Hoffmann A Leung TH Baltimore D Genetic analysis of NF-kappaB/Rel transcription factors defines functional specificities Embo J 2003 22 5530 5539 10.1093/emboj/cdg534 14532125 PMC213788

[B49] Natoli G Saccani S Bosisio D Marazzi I Interactions of NF-kappaB with chromatin: the art of being at the right place at the right time Nat Immunol 2005 6 439 445 15843800 10.1038/ni1196

[B50] Saccani S Pantano S Natoli G Modulation of NF-kappaB activity by exchange of dimers Mol Cell 2003 11 1563 1574 10.1016/S1097-2765(03)00227-2 12820969

[B51] Kunsch C Rosen CA NF-kappa B subunit-specific regulation of the interleukin-8 promoter Mol Cell Biol 1993 13 6137 6146 8413215 10.1128/mcb.13.10.6137-6146.1993 PMC364673

[B52] Moshynska O Sankaran K Pahwa P Saxena A Prognostic significance of a short sequence insertion in the MCL-1 promoter in chronic lymphocytic leukemia J Natl Cancer Inst 2004 96 673 682 10.1093/jnci/djh122 15126604

